# Isolated pulmonary IgG4-related disease mimicking lung malignancy

**DOI:** 10.1259/bjrcr.20160134

**Published:** 2017-04-12

**Authors:** Louise I T Lee, Rebecca Gillibrand, Dev Mukerjee, Sashin S Kaneria

**Affiliations:** Department of Radiology, North Middlesex University Hospital NHS Trust, England, UK

## Abstract

Immunoglobulin G4 (IgG4)-related disease is a relatively rare and only recently recognized immune-mediated fibro-inflammatory condition that is commonly associated with autoimmune pancreatitis. Reports have further been characterized in almost all other organ systems, with several lung-related IgG4 disease reports emerging over the past decade. IgG4-related disease affects more than one organ in 60–90% of patients. To this date, there have been few published cases of pathologically proven isolated IgG4-related lung disease (IgG4-RLD), where no other organ is affected. We report an isolated pulmonary case of IgG4-RLD in a 65-year-old female with clinical and radiological manifestations suggestive of primary lung malignancy. CT revealed multiple sub-solid ground glass opacities, several of which were part-solid, others were pure ground glass. Histological analysis revealed IgG4 disease with no evidence of neoplasia. Serum IgG4 levels were elevated (206 mg dl^–1^). Malignancy was ruled out and the patient was treated with corticosteroids, though there was no change in CT appearance over 16 months. The CT imaging pattern in our case is atypical from previous literature characterisation.

## Case presentation

A 65-year-old female non-smoker of Indian origin living in the UK for 42 years was referred to rheumatology outpatients with a 5-year history of mild arthralgia affecting the shoulders, hands and elbows. A 6-month history of productive cough without haemoptysis and concurrent weight loss was also reported. No fever, night sweats, chest pain or shortness of breath on exertion was present. Examination of the chest, cardiovascular system and abdomen was unremarkable. The only finding was nodal osteoarthritis in the hands with evidence of Heberden’s and Bouchard’s nodes, but no evidence of active synovitis.

## Investigations

Laboratory tests revealed raised ESR 118 mm hr^–1^, CRP 18 mg l^–1^, rheumatoid factor was positive (108 IU ml^–1^), as was ANA at 1/160. ENA, DNA and ANCA were negative.

**Figure 1.**
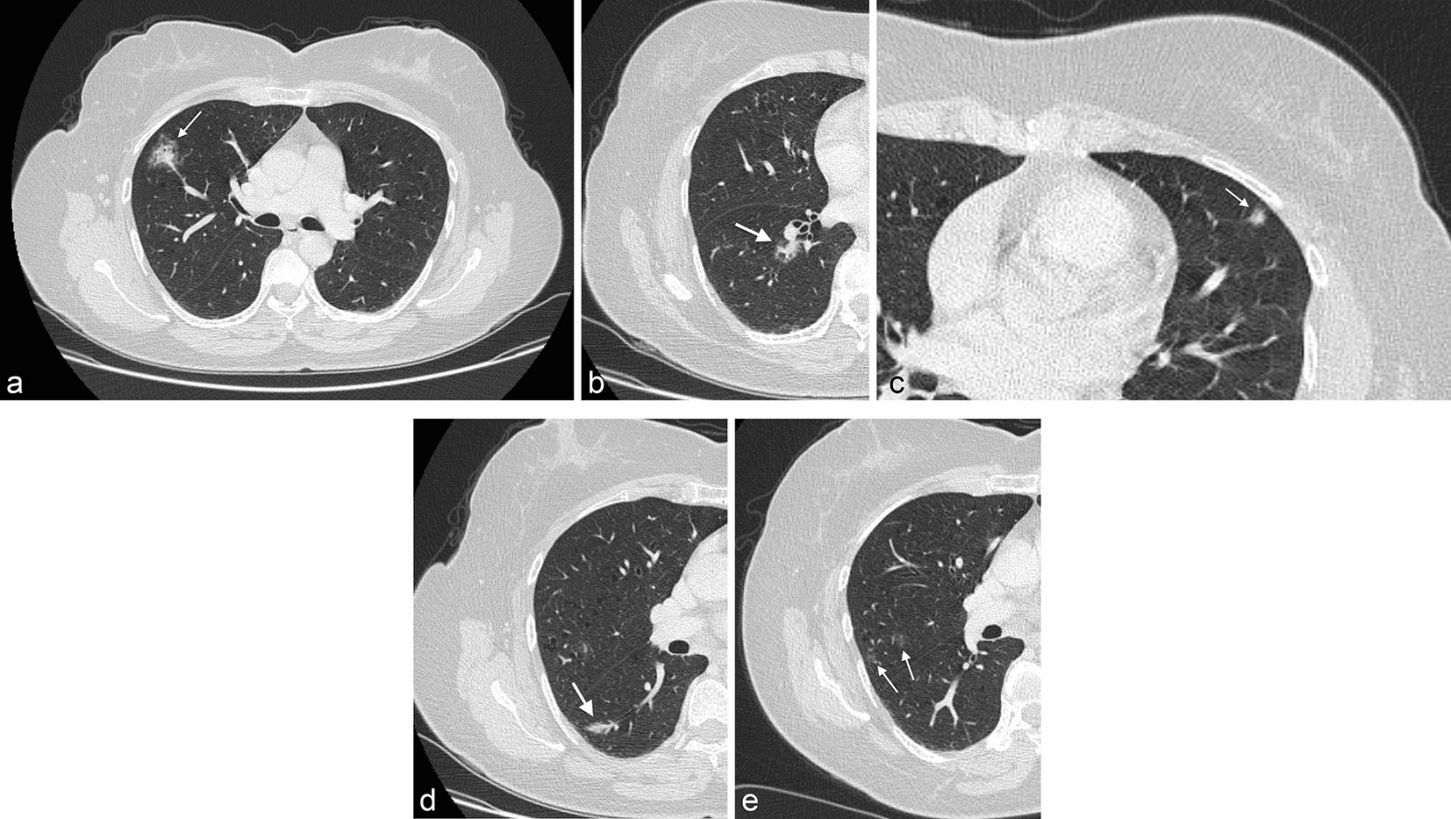
(a–c) CT images showing part-solid opacities with central solid component surrounded by a ground-glass opacity halo. (d) Sub-solid non-round ground-glass opacity. (e) Example of two ill-defined pure ground glass opacities.

CT demonstrated multiple pulmonary sub-solid opacities, many of which were part-solid with a central solid component and a ground-glass halo, others were small pure ground glass nodules (Figure 1). They involved all lobes. There was neither mediastinal or hilar lymph node enlargement present, nor was there any abdominal or pelvic abnormality demonstrated. A repeat CT following a 3-month interval showed these persisted, but had not increased in size or number.

Bronchial washings contained squamous cells, respiratory epithelial cells, mixed leucocytes and blood. No malignant cells were seen. The washings did not grow bacteria or acid-fast bacilli.

**Figure 2.**
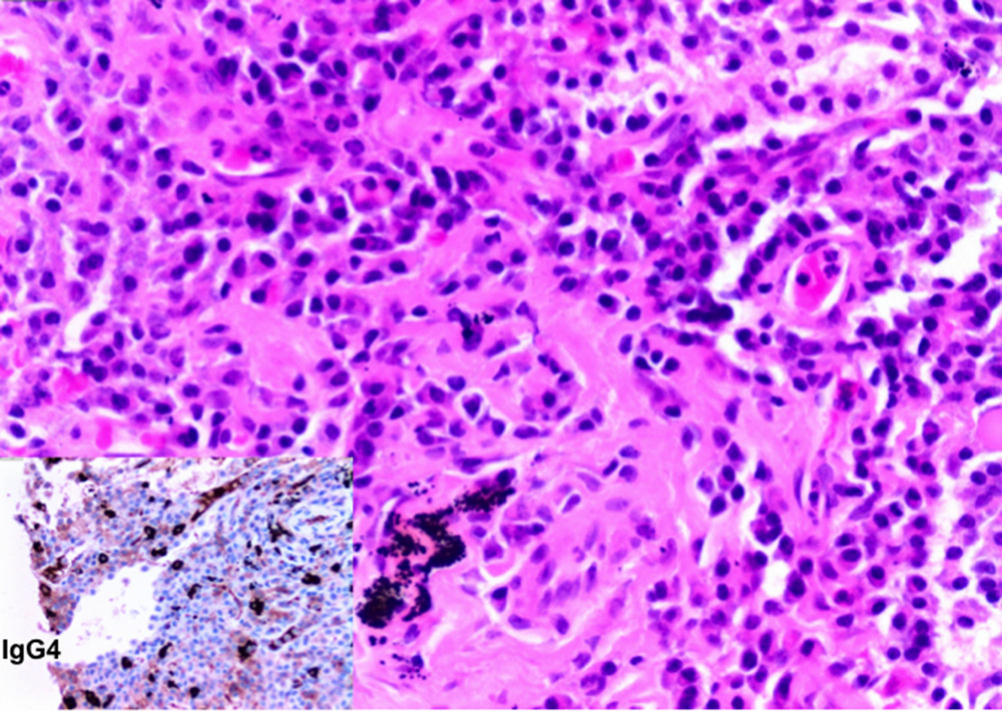
H&E preparation, 200× magnification with image of IgG4 immunohistochemistry (inset). Interstitial fibrosis with lymphoplasmacytic chronic inflammatory infiltrate containing many IgG4 positive plasma cells.

Percutaneous CT-guided lung biopsy of the largest opacity was undertaken. Histological examination revealed lung tissue containing inflammatory cell-rich areas with a fibrous and collagenous background and occasional residual alveoli. Immunohistochemistry confirmed the lymphoplasmacytic nature of the infiltrate with many of the plasma cells expressing IgG4 (more than 50 per high power field) (Figure 2). No evidence of epithelial malignancy was present.

On the basis of histopathological evidence and raised serum IgG4 level (206 mg dl^–1^), a diagnosis of IgG4-related lung disease was made.

## Treatment

Following the diagnosis of IgG4-RLD, the patient was treated with a tapering regimen of prednisolone, commencing at 30 mg once-daily, reducing by 5 mg every two weeks to nil.

## Outcome and Follow-up

Follow-up CT was performed 6 months after starting corticosteroid therapy and a further CT performed at 16 months from the initial presentation. Imaging appearances have been stable, without regression but there are no new lesions. The patient remains clinically well.

## Discussion

IgG4 disease is a recently recognized sclerosing disease characterized by lymphoplasmacytic tissue infiltration with IgG4 plasma cells. Increasing reports show it can involve virtually all organ systems, ranging from the retroperitoneum (archetypically the pancreas), gastrointestinal tract, exocrine and endocrine organs, skin, orbits and lymph nodes. Within the thorax, it can involve lung, mediastinum, pleura and pericardium. Multiorgan involvement is common (>90%), either synchronously or metachronously. It is rare, with an estimated incidence of 0.2 to 1/100,000 in Japan, with no incidence data in other countries.^1–3^

The first pulmonary cases were reported in 2004 in association with autoimmune pancreatitis.^4,5^ Since then, there have been reports of isolated IgG4-related disease in the lung, but precise incidence data has not been established as yet. From the limited cases within the literature, 70–80% appear to affect men of middle to elderly age and most of these cases have been described in the Far East. Approximately half the patients found to have IgG4-RLD presented with no pulmonary symptoms while the rest had non-specific symptoms such as dyspnoea, chest pain or cough.^6,7^

Although elevated serum IgG4 (>140 mg dl^–1^) has been found to be a common occurrence in IgG4-related disease, it is not alone sufficiently sensitive or specific for diagnostic purposes. The level can be normal in 3–30% of affected individuals and can be present in 5% of the normal population, as well as in patients with unrelated conditions including auto-immune disorders.^2,8^

Radiologically, Inoue and colleagues collected 13 cases of IgG4-RLD over 9 years and categorized four major subtypes: (a) solid nodular, with a solitary nodular mass lesion (four cases); (b) round shaped ground glass opacity (GGO), with multiple round GGOs (two cases); (c) alveolar interstitial, with honeycombing, bronchiectasis and diffuse GGO (two cases) and (d) bronchovascular, with thickening of bronchovascular bundles and interlobular septa (five cases).^7^ Most isolated pulmonary cases of pathologically proven IgG4-RLD have described an interstitial disease pattern, either non-specific interstitial pneumonia (NSIP) or usual interstitial pneumonia (UIP). The less common solid nodular and round-shaped GGO can mimic primary lung cancer.^9–11^

IgG4-RLD is still an emerging entity and evidence for ideal management strategy remains scarce. However, there has been recent international consensus statement on the management of IgG4-related disease in general, stating that all symptomatic patients and a subset of asymptomatic patients require treatment.^8^ Glucocorticoids are the first-line agent for active disease unless there are contraindications and some patients may benefit from additional steroid-sparing immunosuppressive agents, including B-cell depletion agents such as Rituximab. Though most cases respond to treatment, relapse is common and progression to multi-organ involvement is also recognised. Nonetheless, a more recent case series reported three pulmonary cases having benign outcomes, with stable disease as well as regression, following conservative management only thus raising the question whether all patients require treatment with immunosuppressive therapy.^12^

Herein we report atypical and interesting findings of our case. Our case somewhat resembles the infrequent round shaped GGO pattern, but unlike Inoue et al. several lesions were part-solid with a ground-glass halo and not all the lesions were round shaped. To the best of our knowledge this is the second only case to describe an associated halo sign and the first in a Western country.^13^ Indeed the prior case by Zhou et al. showed lesions with much more predominant mass like foci with smaller surrounding halo, with clinical presentation more reflective of infection than malignancy. Their biopsy was obtained using fibre-optic bronchoscopy, thus not specifically of lesions with the halo sign, which were commented on to be peripheral. In contrast, our sample was obtained using CT-guided core needle biopsy through the largest lesion with the halo sign itself (Figure 1a). Histologically, normal lung parenchyma was seen to transition into inflammatory cell rich areas with lymphoplasmacytic infiltration, corresponding to the ground glass opacity and a fibrotic region corresponding to the central core consolidative zone. A surgical excision biopsy would aid in providing more detailed architecture for histological evaluation of the halo sign.

In our case, the radiological pattern in combination with weight loss and cough, led to an initial diagnosis of multifocal adenocarcinoma with lepidic growth pattern (formerly classified as bronchoalveolar carcinoma). Given the patient’s medical history of autoimmune disorders, other important differential diagnoses considered were lymphoproliferative disorders, organizing pneumonia or fungal infection. This case highlights the need to maintain an index of suspicion for this albeit rare but increasingly reported condition, which would not be diagnosed without specific histological consideration supported by serum immunoglobulin subtype testing.

## Learning points

1. IgG4-related disease is a recently recognised fibroinflammatory disorder that can affect virtually any organ.
2. Pulmonary manifestations can mimic fibrosis, infection, as well as primary lung malignancy.
3. Diagnosis relies on a radiological imaging, elevated serum IgG4 levels and histopathological confirmation, particularly immunohistochemical staining for IgG4-specific antibody.
4. Little is known about the significance of IgG4-related disease, but it is frequently steroid-responsive so increasing awareness facilitates appropriate testing to establish diagnosis.

## Consent

Written informed consent for the case to be published (including images, case history and data) was obtained from the patient(s) for publication of this case report, including accompanying images.
